# Music Literacy and Soundscape Perception: A Study Based on the Soundwalk Method of Soundscapes

**DOI:** 10.3390/ijerph19148471

**Published:** 2022-07-11

**Authors:** Baoqing Song, Chenyu Gong, Yicheng Gao, Yue Ke, Zehua Wang, Ruichong Lin, Yunji Cai

**Affiliations:** 1 Art Education Center, Beijing Institute of Technology Zhuhai, Zhuhai 519085, China; song_bq@bitzh.edu.cn; 2 School of Computer Technology, Beijing Institute of Technology Zhuhai, Zhuhai 519085, China; 3 Division of Science and Technology, Beijing Normal University-Hong Kong Baptist University United International College, Zhuhai 519087, China; r130233077@mail.uic.edu.cn (Z.W.); r130233046@mail.uic.edu.cn (R.L.); 4 School of Aviation, Beijing Institute of Technology Zhuhai, Zhuhai 519085, China; gongchenyu0203@163.com (C.G.); gyc850929265@163.com (Y.G.); 5 School of Accounting and Finance, Beijing Institute of Technology Zhuhai, Zhuhai 519085, China; keyue8152022@163.com; 6 Student Affairs Office, Beijing Institute of Technology Zhuhai, Zhuhai 519085, China

**Keywords:** aesthetic education, soundscape, higher education, music literacy, campus environment

## Abstract

To explore a method of promoting college aesthetic education through campus environments, the Aesthetic Education Center of the Beijing Institute of Technology Zhuhai (BITZH-AEC) used the soundwalk method of soundscapes to carry out an experiment on students’ soundscape perceptions on campus. Half of the students who participated in the experiment (*n* = 42) had musical instrument learning experience and musical literacy. The research work used conventional statistical analysis methods and “Soundscapy”, newly developed by the British soundscape research team, to process the experimental data. It was found that the soundscape perception evaluation of students with musical literacy was different from that of ordinary students. This included a difference in the overall evaluation of the three experimental areas and a difference in the degree of dispersion of the soundscape evaluation of all six experimental areas. The study also found that there was no correlation between the acoustic noise level and the students’ evaluations of soundscape perception. BITZH-AEC proposes that aesthetic educators should pay attention to the idea of inspiring students to stimulate cultural imagination through soundscape perception.

## 1. Introduction

Aesthetic education is an important part of education that cannot be ignored at any stage of education. The Chinese government released its latest education documents in 2020, requiring schools at all levels to promote aesthetic education reforms and improvements in Chinese students’ mental health and aesthetic literacy. Specific measures include curriculum reform and art practice. In addition, the policy encourages universities to explore the paths of environmental aesthetic education.

Among the various categories of aesthetic education, music education occupies an important position. Active music education not only promotes improvements in aesthetic quality but also plays a positive role in promoting the teaching results of other disciplines [1]. Students can achieve musical literacy through art appreciation or hands-on practice in musical instrument studies. A study from Sydney [2] showed that instrumentalists with higher musical literacy had higher independent aesthetic standards. However, in the process of learning musical instruments, in addition to mastering musical instrument performance skills, will the improvement in musical literacy affect students’ sound perception? Some researchers have conducted similar studies, and the answer was yes [3]. There is evidence that music training can enhance the perceptual judgment of sound elements [4] and can improve sound frequency discrimination [5], and the musical expertise gained during music training is associated with an enhanced ability to detect statistical regularities in auditory stimuli [6]. More related research results are explained in the next section.

In order to provide a basis for the determination of the direction of environmental aesthetic education in colleges and universities, this study began from the perspective of the campus sound environment and used the soundwalk [7] method of soundscapes in the context of aesthetic education to carry out experiments to obtain the answers to the following two questions:

- Are there differences in the perception of soundscapes among students with different levels of musical literacy?
- Are low-noise areas on campus able to receive positive reviews from students?

The article is divided into six sections. Section 2 introduces the background of the research, including the relationship between music and soundscapes, the latest research progress on soundscapes, the application of soundscapes in education, and the previous results of BITZH-AEC related to this research. Section 3 presents the research methods and materials, including an introduction to the experimental environment, experimental procedures, participants, and analytical methods. Section 4 presents the experimental results, including differences in soundscape perception among students with different levels of musical literacy. Section 5 presents the discussion, including an analysis of musical literacy and soundscape perception, a comparison of the findings with other relevant research findings, and a description of the limitations. Section 6 sets out the conclusions.

## 2. Related Background

### 2.1. The Relationship between Music and Soundscapes

Music has a close relationship with soundscapes, and they have a long history. At about 500 BC, Lao Tzu, the founder of Chinese Taoism, once commented the following on the relationship between music and soundscapes: “When Silence Is the Best Music”. This means that the highest aesthetic state of people’s music is to be able to understand the beauty of natural sounds. In *The Tuning of The World* (1977), Schafer opened the doors of soundscape research to modern society, and as a musician and educator, he used moving language to illustrate the importance of soundscape research. The book points out that the home territory of soundscape studies is a middle ground between science, society, and the arts (particularly music) [8]. While the importance of soundscapes to the environment, health, and wellbeing is gradually being recognized by people, scientists and technicians are continuing to study the combination of soundscapes and music. Iyendo suggested that music-infused soundscapes could make patients more emotionally positive in hospitals [9]; playing soothing music was shown to reduce stress, blood pressure, and post-operative trauma when compared to silence in a hospital environment. Carvalho found that soundscapes that incorporated music could change taste feedback [10]. By playing two different sets of music in an environment to bring people different taste experiences, he concluded that sound could have a perceptual effect on the taste of food. Truax added electronic music as part of a soundscape to create composite soundscapes [11]; this work reversed the tradition of listening to ambient soundscapes as music. It is these research efforts that have made music and soundscapes so closely linked.

### 2.2. The Progress of Soundscape Research

In a Web of Science search for highly cited research articles entitled “soundscape” since 2019, it was found that the fields of environmental science, acoustics, and ecology have carried out the most relevant research. In the field of environmental science and acoustics, Kang and his research partners led the formulation and improvement of the ISO 12913 series of standards [7,12,13] and gradually deepened their research on soundscapes and soundscape perception in human settlements [14,15,16,17,18,19]. Among their studies, the evidence obtained from one of the large-scale soundscape surveys [20] showed that pleasantness and eventfulness were significantly different among locations. Their research of UK residents’ noise complaints during the COVID-19 lockdown found that noise complaints (particularly from neighbors) have become even more of a critical factor in the context of public health and people’s wellbeing [21]. The visual analysis toolbox named “Soundscapy” developed by this team in March 2022 makes the results of soundscape research based on ISO standards more intuitive [22]. Jaume Segura-Garcia and his colleagues designed a 5G IoT system for real-time psychoacoustic soundscape monitoring that will be able to compute PA metrics using computational offload based on a proposed functionality split into 5G infrastructure [23]. Their most recent soundscape research in the Water Tribunal of the Plain of Valencia provides information on the applicability of the tools and procedures used in closed spaces for the acoustic study of open and semi-open intangible cultural heritages [24]. Chinese scholars have also conducted research on the soundscapes of cities [25,26,27], temples [28,29], and parks [30,31]. In a soundscape experiment combined with EEG technology in an urban park in Chongqing, China, Xie found that more restorative EEG responses were observed under auditory stimulation than under audiovisual stimulation [32]. Natural sounds can produce more restorative benefits than simply strengthening positive visual aspects.

### 2.3. Soundscapes in Education

The research on the combination of education and soundscapes is not as rich as that in the fields of environmental science, acoustics, and ecology, but there are many educators working in this area. For example, Schoer added soundscapes to history education and established soundscape rooms in museums, enhancing audiences’ perceptions by allowing them to listen to the historical soundscapes of Native Americans in North America [33]. Ikhwanuddin et al. surveyed students’ subjective evaluations of soundscapes in a university library [34]. Milo helped students build soundscape concepts in architectural design classes to promote the ability of these future urban designers to pay more attention to the impact of soundscape factors on the urban environment [35]. Foss and Burgess found in climate education that adding soundscapes to instructional videos increased students’ emotional resonance [36]. D’Alessandro et al. carried out research on a campus outdoor environment soundscape at Roma TRE University. The experiments were conducted in summer and winter. A data analysis showed that students’ perceptions and evaluations of the soundscape were closely related to visual and climatic conditions [37]. Tristán-Hernández et al. conducted a series of studies on campus soundscapes. They found that campus noise affects students’ physical and mental health and academic performance to a certain extent [38]. They studied the effects of background noise in university facilities on brain waves related to concentration and memory in students and employees. Using an integrated research approach based on attention-demanding tasks and EEG electrophysiological assessments, they found that noise exposure was associated with decreased attention [39]. In 2018, they verified through experiments that the noise annoyance evaluation method and the Noise Annoyance Index (PNAI) can effectively evaluate the impact of campus noise on students, help protect students from the impact of noise on physical and mental health, and improve students’ academic performances [40].

### 2.4. Music Education and Sound Perception

Learning to play an instrument is a highly complex task involving the interaction of multiple modalities and higher order cognitive functions. The auditory system is one of the human perceptual systems most affected by musical training [41], which enhances sound processing and cognition, including neural responses to changes in pitch, duration, intensity, and sound-onset timing [42]. The sound features that are important to trained musicians are not limited to music [43]; musicians also have a clear advantage in everyday listening, with studies showing that they can recognize more sound frequencies in noise [44]. In a music training program carried out in a Chicago public high school, it was found that after two years of singing/instrumental training, teenagers could recognize sound frequencies in a noisy environment faster [45], and the simultaneous exercise of fitness training does not boost sound perception. This finding was later replicated and corroborated in younger groups of children [46]. Music players convey emotion to the audience through their musical performance, modulating the acoustic expression of the instrument through their playing skills [47]; in the process, the acoustic feedback is connected with the emotion, meaning that they have different acoustic characteristics from ordinary people and cognitive effects of emotion. There is evidence from many studies that musical ability is positively related to the ability of sound affective perception [48,49].

### 2.5. Preliminary Research Results by BITZH-AEC

BITZH-AEC is in charge of aesthetic education for all students at the college. In 2021, a series of innovative methods of aesthetic education were explored. Among the questionnaires, the results of a question concerning the choice of appreciating art on-campus or off-campus drew attention. Compared with on-campus art appreciation [50], students had a higher demand for off-campus art appreciation [51]. In subsequent interviews, students expressed that they preferred the atmosphere experienced in the off-campus art appreciation process, which included the landscape, soundscape, and the feeling of the relaxing experience brought by the off-campus environment. Among the students who participated in the interviews, some were members of the music club with high musical literacy, and they mentioned the influence of the sound environment more frequently than other students; this was also one of the factors that led the research team to carry out this experiment. In the aesthetic education policy released by the Chinese government in 2020, in addition to the rigid requirements for university art courses, middle schools and primary schools are also required to carry out more art courses for students to improve their aesthetic literacy. This includes the establishment of compulsory art items in the assessment of junior high school and senior high school. Music level is an important part of the assessment of artistic level. From 2024, these music-trained students will enter university campuses. Will they have higher requirements for a school’s soundscape? Is it enough for the campus soundscape to be dominated by quietness? What factors can be used to achieve the purpose of environmental aesthetic education? These questions are worth studying and discussing.

## 3. Materials and Methods

### 3.1. Research Method

This experiment used the soundwalk method in the ISO/TS 12913-2 [7] standard as the main research method. Listening to the entire environment is the core of a soundwalk. After walking along a designated route and listening, a subjective evaluation of the soundscape was carried out by scoring three groups of question scales. The following three groups of questions were included:

- Questions consisting of judgments about local sound elements, including noise, sounds from human beings, and natural sounds (Alternative version).
- Questions about eight perceptual attributes (PAs), namely, pleasant, chaotic, vibrant, uneventful, calm, annoying, eventful, and monotonous.
- Overall impression.

In order to meet the requirements of the ISO/TS 12913-2 standard for soundwalk experiments, the research team printed a paper version of the questionnaire for participants to fill in, and a leader was introduced as a route guide during the soundwalk experiment. In addition, since the Chinese version of the ISO 12913 series of standard documents has not yet been developed, the Chinese version of the PA descriptions used in this study referred to the Chinese part of a soundscape assessment multilingual proposal published at the 49th International Congress and Exposition on Noise Control Engineering [52].

### 3.2. Experimental Participants

Volunteers were recruited from the student associations of BITZH to participate in this experiment. In the recruitment notice, the research team noted that students with musical instrument experience would be given priority. In the end, a total of 42 student volunteers were recruited, aged 18–21, comprising 19 males and 23 females, and 21 of them had musical instrument learning experience, including learning piano, guzheng, guitar, and other musical instruments, with a minimum learning experience of 3 years and a maximum of 15 years; detailed information of the volunteers is shown in Table A1. Using a conventional solfeggio test method, 21 students had good pitch and had a more accurate judgment of the strengths and weaknesses of music, and they were assigned to Team A. The other 21 students were assigned to Team B, and they had no experience of learning a musical instrument and an ordinary level of musical literacy. Two senior students who had over 200 h of recording studio experience participated in the experiment as “local experts”. They were responsible for recording the natural sounds of the campus in the atoms sound material recording work carried out in the school recording studio; thus, they were familiar with the soundscape of the area involved in the soundwalk route.

### 3.3. Case Study Area

**Figure 1:**
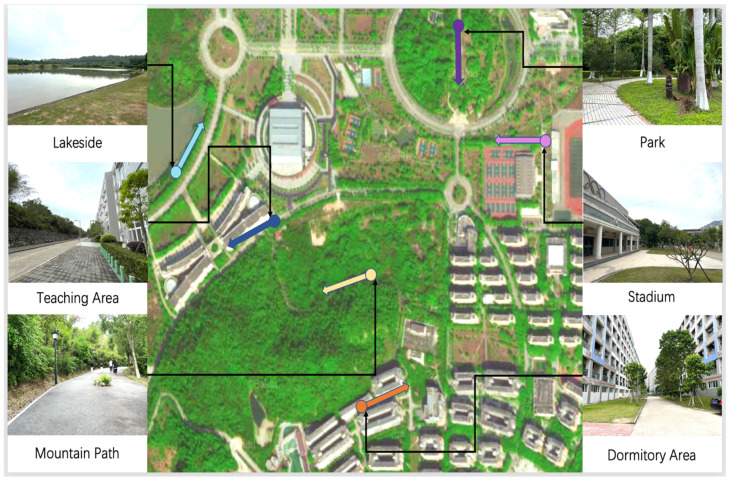
Experimental route.

The case study area was on the BITZH campus, and it included six areas that students often pass through, namely, the dormitory area, a mountain path, a teaching area, a lakeside, a park, and a stadium, as shown in Figure 1. In the figure, the range of the experimental area is marked with different colors, and the soundwalk route is marked with lines; the circle represents the starting point of the soundwalk in the area, and the arrow represents the end.

### 3.4. Experimental Process

First, the leader of the research team conducted face-to-face training for all the experimenters, explaining the basic concept of the soundscape, the meaning of the experiment, and how to fill in the questionnaire, including the interpretation of the questions and answers. Second, local experts led the experimenters to conduct the soundwalk experiments in the six areas. In the experiment, the participants were divided into six groups, and each group included ordinary students and students with musical instrument learning experience. The first local expert oversaw the first group, and the other oversaw the last group. To prevent the walk from being too crowded, there was a distance of 10 m between each group, and the students who walked in the front of each group controlled the distance. During the implementation of the soundwalk, the participants were prohibited from talking, eating, and drinking to reduce human-made noise. Thirdly, they completed the walk on a designated route in each soundwalk area. The group stopped at the endpoint for three minutes to listen carefully to the sound elements and then filled out the paper version of the questionnaire. The first local expert was also responsible for measuring basic acoustic data using type II sound-level meters with a statistical analysis function, model AWA5688, which were calibrated before the experiment started.

### 3.5. Analysis Methods

**Figure 2:**
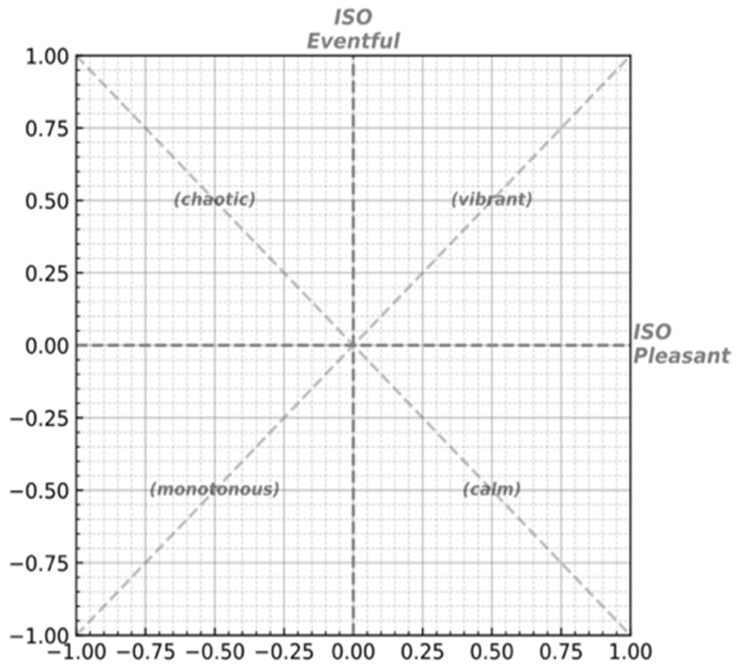
Interface of Soundscapy.

During the analysis, two approaches were used. The first method used conventional statistical analysis methods. Because the sample size was small, and the normal distribution and variance homogeneity did not meet the *t*-test conditions, the Mann–Whitney U test [53] was used to analyze the soundscape feedback data of the two teams of students at the six locations. The results were analyzed to examine whether there were differences in the soundscape evaluations of the two teams. The second method used the latest ISO 8PAs visualization tool named “Soundscapy”, developed by Mitchell, Aletta, and Kang [22]. This tool was used to convert the 8PAs data into two-dimensional ISO coordinates, and they were displayed on a two-dimensional plane with four quadrants in the form of a scatter plot, as shown in Figure 2, so as to more intuitively reflect whether there were differences in the perceptions of the soundscapes between the two teams; refer to the developer’s experimental paper before using this method [54].

## 4. Results

### 4.1. Basic Environmental Conditions

The experiment was carried out on Saturday, 28 May 2022. The school had no teaching courses. Due to the requirements of the COVID-19 epidemic prevention and control, there was a campus closure, and students could not leave the school at will. Before the COVID-19 pandemic, students would go outside campus to carry out various activities on weekends. However, now, because of the inability to carry out rich off-campus activities, more students choose to sleep in till noon. So, during the experiment, there was less movement of people in the areas than during classes. The weather was fine; the wind speed was 4 m/s, the temperature was 28 °C, and the humidity was 70%. The overall feeling was comfortable. To try to ensure that the students participating in the test had a good mental state, the experiment was scheduled to start at 12:00 noon. The experimenters followed the local experts and research teams to carry out the soundwalk experiment in the six different areas according to the predetermined plan. The basic acoustic data recorded using the sound level meter in the six regions are shown in Table 1.

### 4.2. Basic Overview of the Questionnaire Results

**Figure 3:**
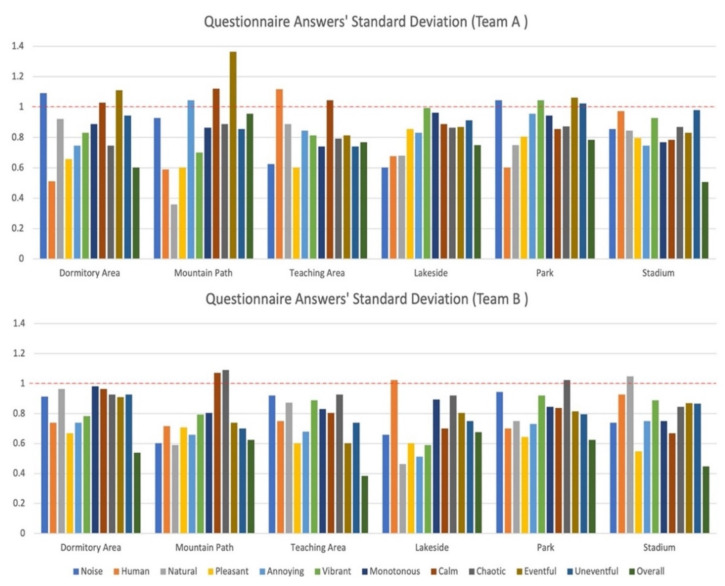
Standard deviations of the two teams’ questionnaire answers.

After a manual verification of the completion of the paper version of the questionnaire, the 252 questionnaires submitted by the 42 subjects were all filled in, and the answers to the paper version of the questionnaire were entered into Excel; the basic data are described in Table 2. It can be seen from this that the medians of the scores of the two teams were different, and, notably, there were 12 items with a standard deviation of more than 1 point for Team A, accounting for 16.7% of the total number of items, while Team B had only 5 items, 6.9% of the total number of items. This shows that the dispersion degree of the PA evaluation carried out by Team A may be greater than that of Team B. This can also be seen intuitively by examining the std results of the two teams’ questionnaire answers using a visualization method, as shown in Figure 3.

### 4.3. Questionnaire Analysis Using the First Method

First, the normality of all the questionnaire answers of Team A and Team B was tested, and the results showed that the two teams met the normal distribution; thus, the next step of the statistical analysis could be carried out. Second, the Mann–Whitney U test was conducted for the answers to the “Overall” question in the overall evaluation of the performance in the six different locations. According to the test results, there were differences in the overall evaluation of the dormitory area, park, and stadium between the two teams of students. In the test of each 8PAs item in each location, the “monotonousness” of the dormitory area, the “calmness” of the park, and the “pleasantness” of the stadium were different. The results are shown in Table 3.

### 4.4. Questionnaire Analysis Using the Second Method

**Figure 4:**
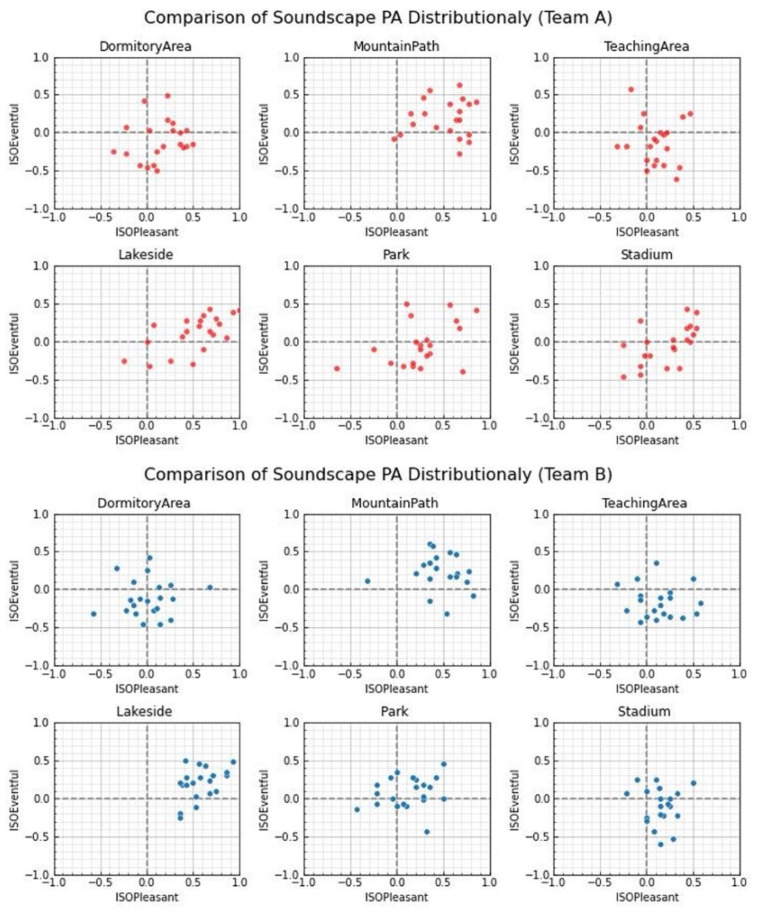
Soundscapy scatterplots.

The results obtained using the second method of analysis were much more interesting. Because this method can make the results more intuitive, one can quickly assess the soundscape perception characteristics of the experimental subjects through the distribution in different quadrants. The combination of multiple Likert responses creates a composite score; this can be considered as a continuous numerical scale, which is appropriate for parametric tests [55]. The soundscape perception 8PAs data of each team of students were converted into a two-dimensional array of soundscape perception features using the calculation tool provided by Soundscapy, and they were used as ISO two-dimensional coordinates, which are presented in the form of scatterplots in Figure 4 For Team A, the degree of dispersion was larger than that of Team B; students who had learned musical instruments had more diverse evaluations of soundscape perception in different places than ordinary students. To further confirm this result, the two-dimensional array was verified using the standard deviation, an evaluation index of data dispersion. The larger the standard deviation, the higher the dispersion of these data. The results of the calculation are shown in Table 4. The standard deviation of Team A was greater than that of Team B at all locations, which also confirms the scatterplot results of Soundscapy.

## 5. Discussion

There was a certain difference in soundscape perception between the students who had learned musical instruments and those who had not learned musical instruments. Combining the noise measurement data, data analysis results, and the results presented in Figure 3, it can be seen that, in areas with small dynamic changes in sound, the two teams of students had obvious differences in soundscape perception. Combined with the actual sound situation on site, the dormitory area and the stadium have continuous mechanical noise from an air conditioner compressor, and the park has continuous high-volume insect sounds of different categories. In such an environment where the main noise source can be clearly identified, the students in Team B had more neutral views, while the students in Team A had richer evaluations, and the evaluations were biased toward chaotic and eventful. This may indicate that, by learning musical instruments, they can identify more categories of sounds. There is no doubt that musical instrument learning can promote improvements in musical literacy and cognitive ability [2,4,5,6,56,57,58].

According to existing research [41,43], this can explain the difference in sound perception ability. That is, it is caused by the different learning times of musical instruments and the different types of monophonic/polyphonic training brought by musical instruments; the reason for the large degree of dispersion of Team A comes from this. This frequency recognition ability that is higher than that of ordinary people can result in more sound elements in the sound scene being heard, leading to richer psychological emotions being stimulated, which is confirmed in the low-dynamic-noise areas (dormitory areas, parks, and stadiums) in this experiment. In addition to allowing people to listen to more sound elements, does higher musical literacy also make people pay more attention to bad sound elements? Perhaps it was for this reason that Schafer started his research on soundscapes.

In China’s aesthetic education reform document, universities are encouraged to explore the promotion of aesthetic education by improving the campus environment. This subtle infiltration method of aesthetic education is understandable, because thinking about music and sounds in nature can add new elements of appreciation and recognition to nature and promote the improvement of aesthetic literacy. Some research teams have tried to introduce musical features into public spaces to promote soundscape shaping, and they achieved feasible results [59,60]. However, when using campus soundscape shaping to explore methods of aesthetic education, physical methods, such as simply reducing noise or increasing sound richness, should not be the only ways. Kant once pointed out that only by visualizing the aesthetic object can the aesthetic subject be pleasant [61]. The cultural imagination behind hearing is not purely dependent on sound; it must relate to vision and other figurative symbols to form overall cultural thinking. Just as the students with different levels of musical literacy in the experiment showed obvious differences in the soundscape evaluation of the stadium, different people listening to the same sound in different environments will form different symbolic associations and connections [62]. Therefore, to a certain extent, not only can a soundscape construct a new auditory field, but the cultural representation and imagination behind it is also the result of the overall operation of the educational system. It is an important part of environmental aesthetic education to inspire students to observe the soundscape and to guide students to stimulate cultural imagination in a beneficial direction through soundscape perception.

The limitation of this study was that the students participating in the experiment were of a single age group. They were all young people aged 18–21 and lacked social cognitive experience and life experience, which limited their evaluation of the soundscapes. In addition, the students had learned various types of musical instruments, and their learning durations were different. Before the experiment, they were only given a simple solfeggio test. This test is only useful for the lower limit of musical literacy. The degree of influence of the high level of musical literacy of the students on the experimental results of the music team is difficult to estimate in this experiment. These limiting factors may be the reasons for the high dispersion of PAs in Team A, but they need to be further confirmed by using a larger sample size.

Due to the differences between eastern and western languages and cultures, the accuracy of the translations used in the questionnaire still needs to be further verified, especially for “vibrant” and “chaotic”. During the experiment, only the solfeggio test was used to divide the subjects into teams; would there be a difference if hearing conditions were measured medically? This experiment only verified music literacy and soundscape perception in China; will the situation in other countries be different? These questions are worthy of further study.

## 6. Conclusions

In the process of promoting the reform of aesthetic education in China, the soundscape shaping of university campuses should be followed up in due course. The experimental results show that students with musical instrument learning experience have differences in soundscape perception from ordinary students. With the improvement in college students’ artistic literacy, especially their music literacy, their evaluation of the campus soundscape will also change. Through the evaluation results of the soundscapes in high-noise and low-noise areas in the experiment, the simple low-noise level was not necessarily related to the students’ enjoyment of the soundscape. When the campus administrators realize the importance of the soundscape to the overall evaluation of the campus environment, they may expect more related studies to emerge on the aspects of soundscapes and students’ psychology, and soundscapes and students’ health. Before that, carrying out art-inspired work so that students’ cultural imagination can be stimulated in a beneficial direction through soundscape perception to better appreciate the “universal concert which is always in progress” is the responsibility of aesthetic educators.

## Figures and Tables

**Table 1:** Acoustic statistics.

Location	L_zeq_.T (dB)	L_Aeq_.T (dBA)	L_Zmax_ (dB)	L_Amax_ (dBA)	L_Zmin_ (dB)	L_Amin_ (dBA)	L_A10_ (dBA)	L_A50_ (dBA)	L_A90_ (dBA)	L_A10_–L_A90_ (dBA)
Dormitory Area	63.0	51.6	81.5	66.5	59.2	49.2	52.8	51.0	50.2	2.6
Mountain Path	56.9	45.5	79.5	62.9	51.9	35.5	47.6	41.2	38.0	9.6
Teaching Area	59.4	49.7	75.5	67.8	54.3	40.2	51.2	44.0	41.4	9.8
Lakeside	58.3	42.9	66.4	59.2	53.0	35.3	45.6	40.4	37.6	8.4
Park	67.2	48.2	75.5	56.8	60.9	45.0	49.5	47.3	46.4	3.1
Stadium	68.0	50.1	82.4	71.3	62.5	44.5	51.8	48.2	46.4	5.4

**Table 2:** Basic questionnaire data of the two teams.

**Basic Questionnaire Data of Team A**												
	**Dormitory Area**		**Mountain Path**		**Teaching Area**		**Lakeside**		**Park**		**Stadium**	
	**Median**	**std**	**Median**	**std**	**Median**	**std**	**Median**	**std**	**Median**	**std**	**Median**	**std**
Noise	3	1.091	1	0.928	3	0.625	1	0.602	3	1.044	3	0.856
Human	2	0.512	1	0.59	3	1.117	1	0.676	1	0.602	2	0.973
Natural	4	0.921	5	0.359	3	0.889	5	0.68	4	0.75	3	0.845
Pleasant	3	0.658	5	0.602	3	0.602	5	0.856	4	0.805	4	0.796
Annoying	2	0.746	2	1.044	2	0.845	2	0.831	2	0.956	2	0.746
Vibrant	3	0.831	4	0.7	3	0.814	4	0.995	3	1.044	3	0.928
Monotonous	3	0.889	2	0.865	3	0.74	2	0.964	2	0.944	3	0.768
Calm	4	1.03	4	1.121	3	1.044	4	0.889	4	0.856	3	0.784
Chaotic	2	0.746	2	0.889	3	0.793	2	0.865	2	0.873	2	0.87
Eventful	3	1.111	4	1.365	2	0.814	4	0.87	3	1.062	3	0.831
Uneventful	3	0.944	2	0.856	4	0.74	2	0.913	3	1.024	3	0.981
Overall	3	0.602	5	0.956	3	0.768	5	0.75	4	0.784	3	0.507
**Basic Questionnaire Data of Team B**												
	**Dormitory Area**		**Mountain Path**		**Teaching Area**		**Lakeside**		**Park**		**Stadium**	
	**Median**	**std**	**Median**	**std**	**Median**	**std**	**Median**	**std**	**Median**	**std**	**Median**	**std**
Noise	5	0.913	1	0.602	3	0.921	2	0.658	3	0.944	3	0.74
Human	2	0.74	2	0.717	2	0.75	2	1.024	2	0.7	2	0.928
Natural	3	0.964	5	0.59	3	0.873	5	0.463	3	0.75	3	1.049
Pleasant	3	0.669	4	0.707	3	0.602	5	0.602	3	0.644	3	0.548
Annoying	3	0.74	2	0.658	3	0.68	1	0.512	2	0.73	3	0.75
Vibrant	3	0.784	4	0.793	3	0.889	4	0.59	3	0.921	3	0.889
Monotonous	4	0.981	2	0.805	3	0.831	2	0.894	3	0.845	3	0.75
Calm	3	0.964	3	1.071	3	0.805	4	0.7	3	0.837	3	0.669
Chaotic	3	0.928	2	1.091	2	0.928	2	0.921	3	1.024	2	0.845
Eventful	3	0.91	4	0.74	2	0.602	4	0.805	3	0.814	3	0.87
Uneventful	3	0.928	2	0.7	4	0.74	2	0.75	3	0.796	3	0.865
Overall	3	0.539	4	0.625	3	0.384	5	0.676	3	0.625	3	0.447

**Table 3:** Mann–Whitney U test *p*-value results of the two teams.

PA Item	Dormitory Area	Mountain Path	Teaching Area	Lakeside	Park	Stadium
Overall	0.049	0.558	0.125	0.711	0.042	0.008
Pleasant	0.096	0.024	0.972	0.620	0.201	0.004
Annoying	0.660	0.218	0.283	0.086	0.713	0.660
Vibrant	0.871	0.782	0.593	0.421	0.591	0.368
Monotonous	0.049	0.578	0.830	0.707	0.936	0.695
Calm	0.234	0.230	0.518	0.945	0.011	0.602
Chaotic	0.137	0.779	0.197	0.788	0.041	0.621
Eventful	0.512	0.895	0.956	0.073	0.771	0.316
Uneventful	0.187	0.576	0.988	0.668	0.270	0.508

*p*-value < 0.05 indicates the difference between the two teams’ data, which is highlighted in gray in the table.

**Table 4:** Standard deviations of two-dimensional ISO coordinate values.

	Dormitory Area	Mountain Path	Teaching Area	Lakeside	Park	Stadium
Team A	0.275	0.281	0.271	0.340	0.341	0.282
Team B	0.252	0.256	0.268	0.273	0.227	0.241

## Data Availability

The datasets generated during the current study are available from the corresponding author on reasonable request.

## Appendix A

**Table A1:** Basic information of subjects.

**Team A**					**Team B**		
**No.**	**Gender**	**Age**	**Instrument**	**Duration**	**No.**	**Gender**	**Age**
A01	Female	19	Guzheng	3	B01	Female	19
A02	Female	19	Guzheng	10	B02	Male	20
A03	Female	18	Guitar	3	B03	Female	20
A04	Male	19	Piano	12	B04	Male	20
A05	Female	19	Violin	3	B05	Male	19
A06	Female	19	Piano	13	B06	Male	19
A07	Male	19	Piano	10	B07	Male	21
A08	Male	18	Sax	7	B08	Female	21
A09	Male	20	Guitar	6	B09	Male	21
A10	Male	20	Guitar	4	B10	Female	20
A11	Female	20	Piano	15	B11	Female	21
A12	Female	19	Guzheng	11	B12	Male	20
A13	Female	19	Guitar	5	B13	Male	20
A14	Male	20	Piano	3	B14	Female	19
A15	Female	19	Guitar	4	B15	Female	20
A16	Female	20	Guzheng	5	B16	Male	21
A17	Male	20	Flute	6	B17	Female	20
A18	Female	21	Guzheng	7	B18	Female	20
A19	Male	21	Guitar	6	B19	Male	20
A20	Female	21	Piano	9	B20	Female	19
A21	Male	20	Yangqin	11	B21	Female	20

**Table A2:** Main contents of the soundwalk questionnaire (English and Chinese).

请为你听到的每种声音选择一个响应选项 Please Tick off One Reponse Alternative Per Type of Sound	完全没有 Not at All	一点 A Little	有且适度的 Moderately	许多 A Lot	明显且突出 Dominates Completely
噪声 ( 交通声、机械声等) Noise (e.g., traffic, construction, industry)	□	□	□	□	□
人类声音 (嬉笑、交谈、脚步声等) Sounds from human being (e.g., conversation, children at play, footsteps)	□	□	□	□	□
自然声 (鸟叫、植物摩擦、水声、虫声等) Natural sounds (e.g., singing birds, flowing water, wind in vegetation)	□	□	□	□	□
请对周围声音环境从8个方面选择符合你的感受的选项 For each of the 8 scales below, to what extend do you agree or disagree that the present sourrounding sound environment is...	非常同意 Strongly agree	同意 Agree	既不同意, 也不反对 Neither agree, nor disagree	不同意 Disagree	非常不同意 Strongly disagree
令人愉快的 pleasant	□	□	□	□	□
纷乱繁杂的 chaotic	□	□	□	□	□
充满活力的 vibrant	□	□	□	□	□
平淡无奇的 uneventful	□	□	□	□	□
平静的 calm	□	□	□	□	□
烦人的 annoying	□	□	□	□	□
多彩丰富的 eventful	□	□	□	□	□
单调乏味的 monotonous	□	□	□	□	□
	很好 Very good	好 Good	不好不坏 Neither good, nor bad	不好 Bad	非常糟糕 Very bad
总的来说, 你会如何评价当前的声音环境? Overall, how would you describe the present surrounding sound environment?	□	□	□	□	□
